# Combination of biochemical and mechanical cues for tendon tissue engineering

**DOI:** 10.1111/jcmm.13186

**Published:** 2017-05-04

**Authors:** Stefano Testa, Marco Costantini, Ersilia Fornetti, Sergio Bernardini, Marcella Trombetta, Dror Seliktar, Stefano Cannata, Alberto Rainer, Cesare Gargioli

**Affiliations:** ^1^ Department of Biology Tor Vergata Rome University Rome Italy; ^2^ Department of Engineering Università Campus Bio‐Medico di Roma Rome Italy; ^3^ Department of Biomedical Engineering Techion Institute Haifa Israel

**Keywords:** artificial tendon, tissue engineering, mechanical stimulation, biomaterial, bioreactor

## Abstract

Tendinopathies negatively affect the life quality of millions of people in occupational and athletic settings, as well as the general population. Tendon healing is a slow process, often with insufficient results to restore complete endurance and functionality of the tissue. Tissue engineering, using tendon progenitors, artificial matrices and bioreactors for mechanical stimulation, could be an important approach for treating rips, fraying and tissue rupture. In our work, C3H10T1/2 murine fibroblast cell line was exposed to a combination of stimuli: a biochemical stimulus provided by Transforming Growth Factor Beta (TGF‐β) and Ascorbic Acid (AA); a three‐dimensional environment represented by PEGylated‐Fibrinogen (PEG‐Fibrinogen) biomimetic matrix; and a mechanical induction exploiting a custom bioreactor applying uniaxial stretching. *In vitro* analyses by immunofluorescence and mechanical testing revealed that the proposed combined approach favours the organization of a three‐dimensional tissue‐like structure promoting a remarkable arrangement of the cells and the neo‐extracellular matrix, reflecting into enhanced mechanical strength. The proposed method represents a novel approach for tendon tissue engineering, demonstrating how the combined effect of biochemical and mechanical stimuli ameliorates biological and mechanical properties of the artificial tissue compared to those obtained with single inducement.

## Introduction

Tendon is a peculiar connective tissue connecting and transferring energy from muscle to bone, providing stability and mobility to the entire body. This connective tissue is complex and organized in a hierarchical way. Structurally, the tendon basic units are the collagen fibrils, which are synthesized starting from soluble tropocollagen molecules. Collections of these fibrils constitute bundles, arrays of bundles form fascicles and, finally, a group of fascicles get together to form a tendon. Each fibril bundle is surrounded by a thin sheath of connective tissue, namely endotenon, while vessels and nerves are located between the endotenon and the epitenon, a more external membrane which encompasses several bundles 1.

Mature tendon tissue appears hypocellular and hypovascular, composed of an abundant extracellular matrix (ECM) produced by resident tenoblasts and tenocytes. The main component of tendon ECM is collagen, which represents 65–80% of the total dry mass of tendon (type I collagen representing 95% of total, with type III collagen being the second most abundant). Due to the presence of collagen fibres, and especially to their organization into parallel bundles, tendons are able to withstand very high tensile and torsion forces 1, 2, 3. However, following trauma or excessive load, they are subjected to rip, fraying or rupture.

Nowadays, tendinopathies represent major medical issues associated with physical activity and age‐related degeneration. It has been estimated that tendon, ligament and joint capsular pathologies represent 45% of the annual musculoskeletal compartment disorders in the United States 4. Unfortunately, due to hypocellularity and hypovascularity, the natural healing ability of tendons is extremely limited and slow (the healing process may last from few months to one or two years) and the currently available medical treatments often fail in obtaining a recovered tissue with the same characteristics of the native one 5, 6. Overall, the entire healing process can be divided into three distinct phases: (*i*) inflammatory; (*ii*) proliferative; (*iii*) maturation and remodeling. Each phase is driven by different cell populations and several inflammatory proteins and growth factors. Some of these factors, including TGF‐ β, remain constant throughout the process, highlighting their key healing role 7, 8.

For these reasons, the development of new technologies to build a complete and functional tendon is a research field in continuous expansion. So far, one of the most promising strategies is represented by the use of biomimetic matrix scaffolds (preferably hydrogels) capable of mimicking the extracellular matrix and providing a support for the growth, differentiation and organization of tenocyte precursors 9, 10. Such precursors, as demonstrated in the recent past, may be harvested from different sources including periosteum 11, 12, 13, bone marrow 14, 15, 16, 17, tendon itself 16, 17, 18 and adipose tissue 4, 17. Different biomaterial‐based systems have been developed and tested for the fabrication of artificial tendon tissue, including natural matrices 19, 20, 21, hybrid matrices (featuring natural and synthetic components) 22, 23 and fully synthetic matrices 14, 24. However, cells and biomaterials alone are not sufficient to achieve an optimal level of differentiation and organization. In fact, a key role is played by growth factors, able to provide transcriptional activation of genes involved in tenogenic differentiation 8, 25. Among these, TGF‐β has been shown to promote the production and incorporation of collagen and other ECM components in the matrix itself 26, 27. Mechanical stimulation is another pivotal factor for tenogenic differentiation. It has been demonstrated that mere cyclic mechanical stimulation of cells embedded in a three‐dimensional support is able to activate tenogenic pathways 14. For this reason, several studies have been performed to assess the effect of cyclic mechanical stimulation on tenogenesis, demonstrating its crucial role for the development of a well‐structured artificial tendon tissue, with correct ECM orientation 17, 23, 28, 29, 30, and several research groups have documented the design of bioreactors for tendon tissue engineering 31, 32.

In this work, we treated the murine fibroblast cell line CH310T1/2 with TGF‐β and Ascorbic Acid (AA), an important cofactor for tenogenic differentiation 33. Cells were grown in a 3D environment represented by PEG‐Fibrinogen (PF) 34, a semisynthetic hydrogel matrix that has been shown to provide good support for cell growth and differentiation in other settings, such as skeletal muscle regeneration 35, 36, 37. Cell‐laden PF hydrogels were cultured using a purposely designed bioreactor which conveniently applied cyclic uniaxial stretch to hydrogel constructs.

## Materials and methods

### Cell culture

C3H10T1/2 (10T/2) cells were cultured on conventional Petri dishes (BD Falcon NY, USA) at 37°C, 5% CO_2_ in DMEM GlutaMAX (Gibco MA, USA.) supplemented with 10% heat‐inactivated foetal bovine serum (FBS, EuroClone, Pero (MI), Italy), penicillin (100 IU/ml; Gibco) and streptomycin (100 mg/ml; Gibco). All the experiments performed with 10T/2 in 2D were conducted for 15 days and cells were divided into two experimental groups: the control group (2DC), cultured in growth medium, and the treated group (2DT), cultured in differentiation medium. Differentiation was induced 24 hrs after cell plating by supplementation with TGF‐β 1 (PeproTech London, UK) to a final concentration of 5 ng/ml and ascorbic acid (AA; Sigma‐Aldrich Milan, Italy) to a final concentration of 50 μg/ml. Medium was changed twice a week.

### 3D hydrogel preparation

PF was synthesized as described elsewhere 34. 10T1/2 were resuspended into a 14 mg/ml PF solution in PBS containing 0.1 wt.% photoinitiator (Irgacure 2959, Ciba Specialty Chemicals) at a density of 2.5 × 10^7^ cells/ml. Aliquots (120 μl) of the suspension were poured into strip‐shaped Teflon moulds (10 × 3.3 × 1.5 mm) and cured under a longwave UV lamp (365 nm, 4–5 mW/cm^2^) for 5 min. into a laminar flow hood. After UV crosslinking, samples were immediately transferred into multiwell plates in DMEM growth medium and cultured for 15 days. A control group (3DC) was cultured in growth medium, while a treated group (3DT) was cultured in differentiation medium (as previously described) after the first 24 hrs. Medium was changed twice a week.

### Bioreactor and mechanical stimulation

**Figure 1:**
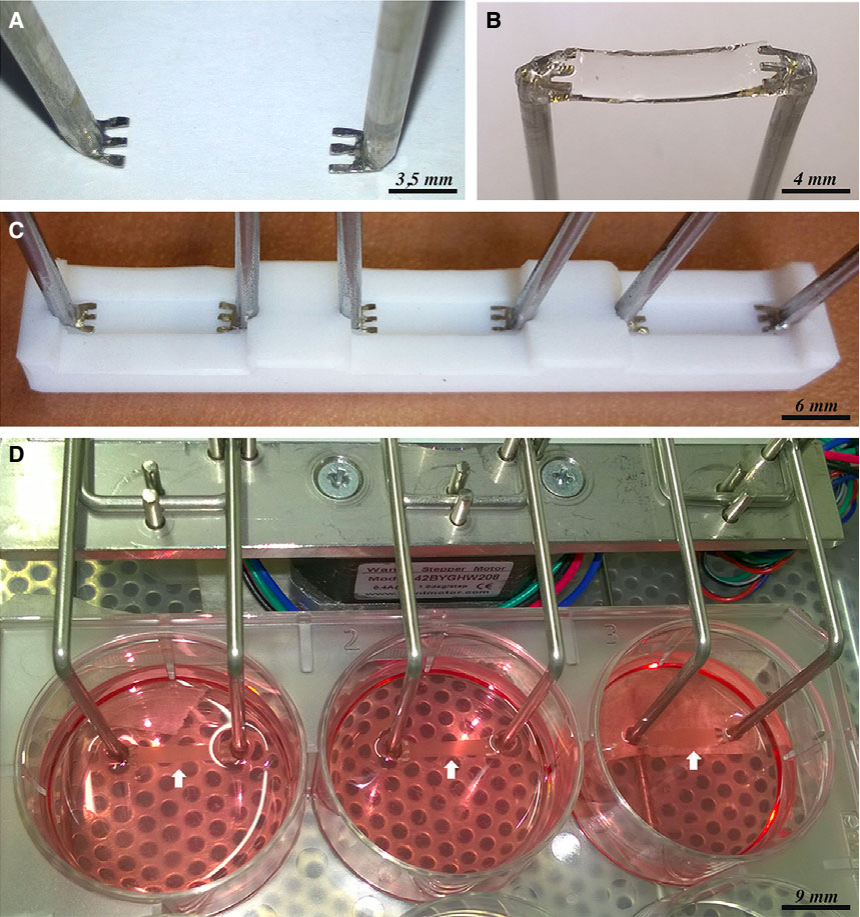
Bioreactor. Comb‐shaped stainless steel pins (**A**); PF hydrogel anchored to the pins (**B**); pins positioned into bar‐shaped Teflon moulds (**C**); one side of the device with the three constructs in position (white arrows) within the 6‐well plate (**D**).

To provide a mechanical stimulation to the cell‐laden PF hydrogels, a custom bioreactor was designed and manufactured. The system was assembled using standard laboratory supplies, with the aim of fabricating a cost‐effective and easy‐to‐use device. Bioreactor was dimensioned to fit 6‐well plates culture supports (Fig. 1D). Among its features, the apparatus enables the application of uniaxial cyclic stretching to six hydrogel constructs simultaneously, with programmable frequency, amplitude (applied strain) and duty cycle.

In particular, cyclic stimulation with a 3.3% duty cycle^1^ on a 60‐min. period, at a stretching frequency of 0.5 Hz and at 10% imposed strain was selected. The anchoring of the hydrogel to the bioreactor was ensured by means of comb‐shaped stainless steel pins (3.5 mm wide, 3 mm long, 0.3 mm thick) which ensured a strong grip (Fig. 1A). To achieve firm attachment of the hydrogel constructs to the pins (Fig. 1B), these were placed into the above described Teflon moulds where cell‐containing PF solution was poured and UV‐crosslinked. The assembly was then fitted to the bioreactor, three constructs per side (Fig. 1D). Bioreactor actuation was provided by a stepper motor controlled by an Arduino Uno [www.arduino.cc] microcontroller board. Mechanical stimulation was applied starting from 24 hrs after polymerization. Also in this case, constructs were divided in two groups: a control group (3DSC) in growth medium and a treated group (3DST) with TGF‐β and AA supplementation.

### Immunofluorescence analysis

Immunofluorescence analysis was performed according to Scardigli and collaborators 38. Briefly, cells and cell‐laden PF constructs were fixed with 2% PFA in PBS for 30 min. at 4°C. Then, samples were washed with PBS and blocked with 10% goat serum in PBS for 1 hr at room temperature (RT). Subsequently, they were incubated with anti‐Collagen Type I primary antibody (rabbit polyclonal, #ab21286, 1:100 dilution; Abcam Cambridge, UK) followed by incubation with AlexaFluor 488 conjugated goat anti‐rabbit IgG (H+L) (Thermo Fisher Scientific #A‐11008, 1:300). Finally, nuclei were counterstained with 300 nM DAPI (Thermo Fisher Scientific MA, USA) for 10 min. Specimens were viewed under a Nikon TE 2000 epifluorescence microscope equipped with a Photometrics CoolSNAP MYO CCD camera.

### Live/Dead assay

Cell viability of 3D constructs was assessed by the use of Cellstain Double Staining Kit (Sigma‐Aldrich), which allows the simultaneous fluorescence staining of viable and death cells. Briefly, after incubation of constructs with Calcein‐AM (viable cells) and Propidium Iodide (dead cells) solutions for 30 min. at 37°C, live and dead cells were counted from fluoresence micrographs. At least three randomly chosen non‐overlapping fields at 10× magnification were acquired for each sample, and the experiment was conducted in triplicate; viability was expressed as the percentage of live cells on total (Fig. S1).

### Quantitative Real‐time PCR (qRT‐PCR)

RNA was extracted from dishes and cell‐laden PF constructs using TRIzol (Thermo Fisher Scientific), according to the manufacturer's instructions. Two micrograms of RNA were retro‐transcribed using the High‐Capacity cDNA Reverse Transcription Kit (Thermo Fisher Scientific), according to the manufacturer's instructions. Quantitative PCR was performed with a real‐time PCR thermocycler (LightCycler^®^, Roche, Monza, Italy). Each cDNA sample was amplified in triplicate using KAPA SYBR^®^ FAST qPCR kit Master Mix (Kapa Biosystems). After verifying the stable expression of glyceraldehyde 3‐phosphate dehydrogenase (GAPDH), this gene was selected as an endogenous control. Relative mRNA levels were calculated by the delta‐delta CT method, according to Livak and colleagues 39. Amplification efficiency for the analysed genes was calculated according to Pfaffl and collaborators 40, while primer specificity was confirmed by melting curve analysis.

Primers used are listed below:

GAPDH (109) – Fw: CGACTTCAACAGCAACTC Rv: GTAGCCGTATTCATTGTCAT
COL1A1 (149) – Fw: GCATTCACCTTTCAAACTTAGT Rv: CTTCAAGCAAGAGGACCAA
COL3A1 (137) – Fw: CAACGGTCATACTCATTC Rv: TATAGTCTTCAGGTCTCAG

For each culture condition (*i.e*. 2D, 3D static and 3D under stretching, gene expression levels were expressed as the ratio between TGF‐β/AA‐treated and non‐treated (control) groups.

### Mechanical testing

Mechanical properties of the constructs (control and treated) either cultured in static conditions (3D group) or by the application of cyclic stretching (3DS group) were assessed by tensile testing (Instron model 3365 with BlueHill software, equipped with a 10 N load cell). Additional control groups were represented by no‐cell hydrogels and freshly seeded constructs.

**Figure 2:**
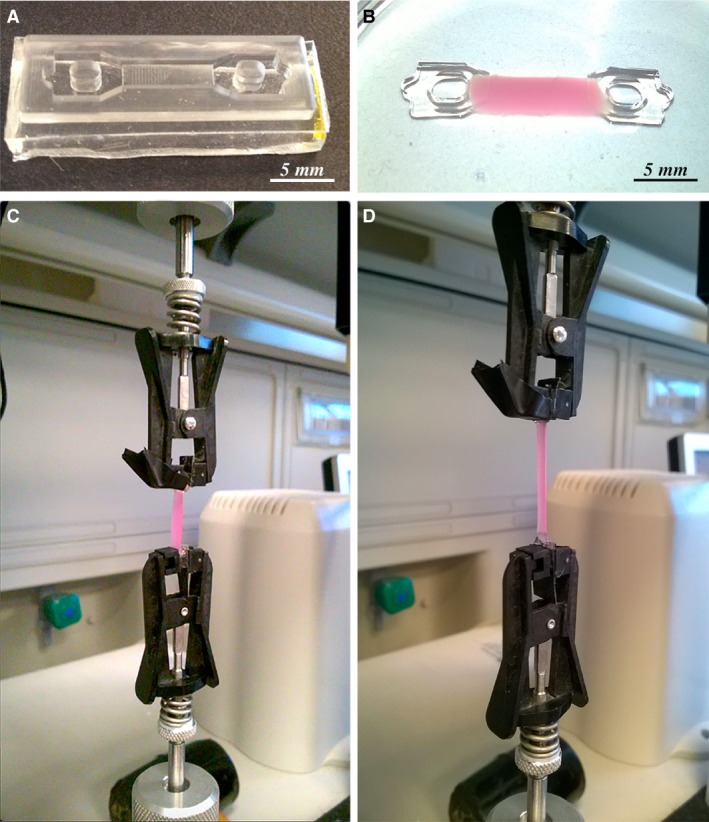
Mechanical tests. Silicone rubber mould used to produce dog‐bone specimens starting from the PF‐cells constructs (**A**); detail of the specimen showing the construct with tapered PEG‐DA ends (**B**); specimen connected tester clamps at start (**C**); specimen at maximum elongation (**D**).

Constructs were placed in the central portion of a silicone rubber mould showing tapered ends (Fig. 2A). A 10 wt.% PEG diacrylate solution (PEG‐DA; Sigma‐Aldrich) containing 0.1 wt.% Irgacure 2959 photoinitiator was poured into the side portions of the mould and UV cured for 5 min., embedding the construct into a ‘dog‐bone’ shaped specimen, suitable for tensile testing (Fig. 2B).

Specimens were individually measured and clamped to the tensile tester by means of spring‐loaded clamps (Fig. 2C). A strain rate of 1.25 × 10^−2^/s was selected and test was conducted up to 30% strain level, while recording the stress–strain curve. Elastic modulus was calculated as the slope of the linear portion of the curve in the 0–10% tract.

### Statistical analysis

All experiments were performed in quintuplicate (*n* = 5). One‐way analysis of variance (anova) was used for multiple means comparisons, followed by *post hoc* testing (Tukey). Statistical significance was at the 0.05 level.

## Results

### Immunofluorescence microscopy

**Figure 3:**
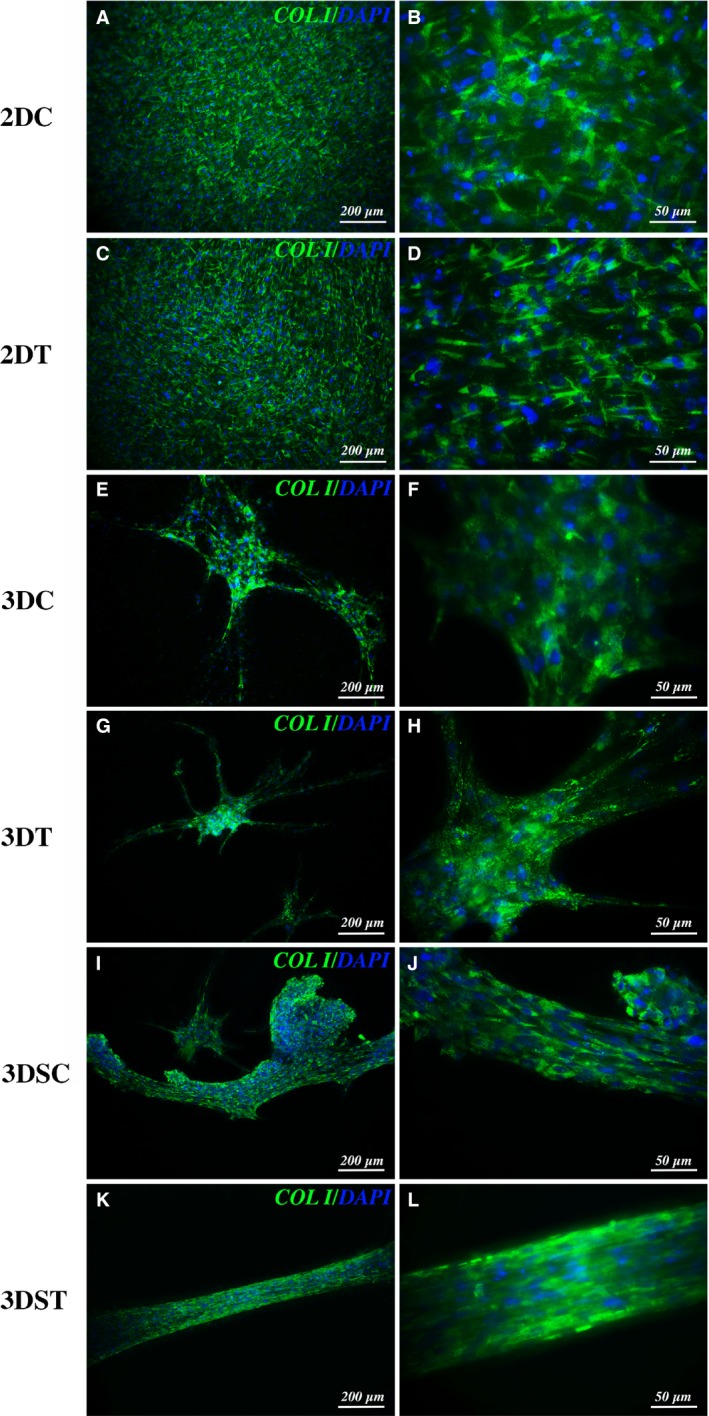
Immunofluorescence analysis. Collagen type I (green) and DAPI (blue) immuno‐staining of 10T1/2 after 15 days for: 2D culture of control (**A, B**) and treated (**C, D**) groups; 3D culture of control (3DC,** E, F**) and treated (3DT,** G, H**) groups under static conditions; 3D culture of control (3DSC,** I, J**) and treated (3DST,** K, L**) groups under mechanical stretching.

After 15 days, adherent cultures in growth medium (2DC) or in differentiation medium enriched with TGF‐β and AA (2DT), were analysed for immunofluorescence against type I collagen, main constituent of tendon fibrils. Results revealed that cell morphology and collagen fluorescent signal of the control group (Fig. 3A and B) were comparable to those of the treated group (Fig. 3C and D).

In a similar manner, analysis of 3D constructs under static culture conditions showed comparable extracellular matrix organization and collagen fluorescence in the control (Fig. 3E and F) and in the treated groups (Fig. 3G and H). Three‐dimensional culture system highlighted remarkable differences in terms of cell distribution compared with 2D adherent cultures, revealing cell clusterization due to tenogenic differentiation, with consequent construct shrinkage and 3D scaffold remodelling (Fig. 3E‐H).

Mechanical stretching was applied to constructs cultured in growth (3DSC) and in differentiation medium (3DST) for 15 days using a purposely developed bioreactor (Video S1). Immunofluorescence analysis results showed an ameliorated matrix organization (Fig. 3I and J) compared to static control (3DC group). In the treated group (3DST), a much more evident degree of organization and deposition of collagen fibres along the stretching direction could be observed (Fig. 3K and L). Live/dead essay revealed a comparable cell survival among the different tested group (15 days of culture or stimuli), with a slight increased death rate upon mechanical inducement (Fig. S1). Moreover, nuclei labelled by 4′,6‐diamidino‐2‐phenylindole (DAPI) staining clearly revealed the effect of mechanical inducement in culture structure organization, promoting a considerable alignment of the PF‐encapsulated and stretched cells towards the straining direction, as underlined by the flattened shape of cell nuclei in the same direction in the treated group (3DST) (Fig. S2).

### Collagen expression levels

**Figure 4:**
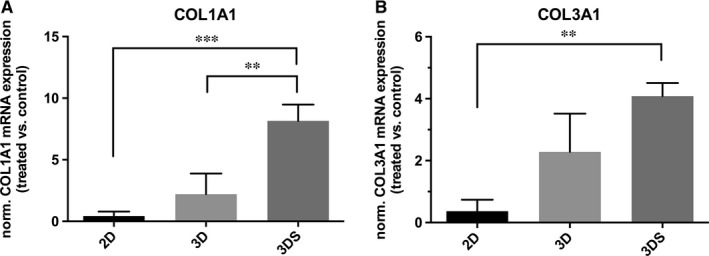
Relative mRNA expression on artificial tendon‐like tissue matrix components. Expression of COL1A1 (**A**) and COL3A1 (**B**) for adherent (2D), 3D static (3D) and 3D stretched (3DS) conditions, expressed as the ratio between treated (TGF‐β/AA) and non‐treated (control) groups. * *P* < 0.05; ** *P* < 0.01; ***P < 0.001.

Figure 4 shows the results of type I and type III collagen expression as analysed by RT‐qPCR, expressed as the ratio between treated (TGF‐β/AA) and non‐treated (control) groups for each culture condition (2D, 3D static, and 3D dynamic conditions).

2D cultures showed a non‐significant decrease in the expression of both collagen isoforms compared to their internal controls. This result is in line with previous evidence, reporting a drop in collagen synthesis under TGF‐β stimulation for fibroblast cells cultured in monolayer 41 and lower collagen (type II) expression for 10T1/2 monolayers during TGF‐β‐induced chondrogenesis 42, and evidence the importance of three dimensional culture settings to promote cell differentiation.

In 3D static cultures, treated constructs showed comparable mRNA expression of type I and type III collagen, with a slight—but not significant—increase compared to control group. In mechanically stimulated constructs, on the contrary, treatment resulted in increased production of both collagen isoforms, with a significantly higher prevalence of type I collagen.

Comparing 3D dynamic culture conditions with 3D static and 2D ones, it was observed that the normalized (TGF‐β/AA‐treated *versus* non‐treated control) expression of collagen I was significantly higher in 3DS group compared to both 2D and 3D groups; 3DS also showed higher normalized collagen III expression compared to 3D group.

### Mechanical characterization

After 15 days of culture, 3D static (3DC, 3DT) and stretched (3DSC, 3DST) constructs were characterized in terms of their mechanical properties; no‐cell (NC) and freshly seeded constructs at time zero (T0, just after embedding) were used as controls to verify the stress–strain behaviour of the PF matrix, revealing a very low elastic modulus for pristine PF, while T0 showed a slightly higher modulus (Fig. S3).

**Figure 5:**
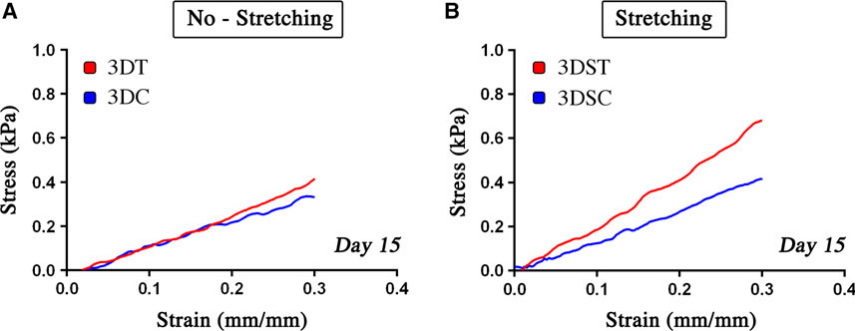
Stress–strain curves. Stress–strain curves of control (3DC) and treated (3DT) groups for constructs after 15 days of static culture (**A**) and of control (3DSC) and treated (3DST) groups for constructs under stretching (**B**).

The elastic modulus of the constructs grown in different conditions was calculated for the initial linear portion of the stress–strain curves (Fig. 5 and Table 1). Static culture groups (3DC and 3DT) had almost the same modulus, while the stretched constructs treated with TGF‐β and AA (3DST) showed an increased elastic modulus compared to the controls (3DSC). Nevertheless, it must be noticed that although the treated and stretched (3DST) constructs presented a higher elastic modulus (2.15 kPa compared to the initial 1.11 kPa), is still distant from the native tendon modulus.

**Table 1:** Elastic modulus

Sample	Modulus (kPa)
3DC	1.37
3DT	1.18
3DSC	1.32
3DST	2.15

## Discussion

The healing process of tendon is very slow and can be divided in three distinct phases, each with its main actors. In the first stage (inflammatory phase), in addition to immune system cells and inflammatory molecules, several growth factors are involved, including TGF‐β. This growth factor is also involved during the second (proliferative), and the third (remodeling) phases 8. Such a persistent presence reveals a pivotal role of TGF‐β in the healing process and in the formation of new tendon tissue. Indeed, its importance in the induction and guidance of tenogenic processes has been well documented: cultures of human fibroblasts extracted from anterior cruciate ligament and grown under mechanical stimulation are able to produce TGF‐β, which in turn stimulates the expression of genes typical of tendon extracellular matrix such as type I and type III collagen. In fact, blocking the action of TGF‐β with specific antibodies drastically inhibits the expression of collagen genes 43. Moreover, vacuum‐based stretching induction on artificial structures containing avian flexor tendon cells (Bioartificial Tissue, BAT), was shown to promote cell alignment arrangement along strain direction with the maintenance of collagen Type I and Type III expression in the engineered artificial tendon tissue 44. In this work a multipotent murine fibroblasts line, the 10T/2, when properly stimulated may undertake various differentiation processes such as osteogenesis, adipogenesis and chondrogenesis 45, was chosen as a model to test the efficiency of the combined administration of TGF‐β and AA in the stimulation of tenogenic fate, as previously documented 25. The use of TGF‐β on this cell line has been shown to activate the tenogenic differentiation process 46. However, in our experiments we observed that TGF‐β and AA alone are not able to significantly promote this differentiation destiny in 2D and 3D cultures. Instead, the synergic action of biochemical and mechanical stimulation proved to be promote a remarkable enhancement of the tenogenic process that led to the formation of an extracellular matrix rich in type I collagen, properly oriented along the stretching direction (Fig. 3K and L). As reported in the literature, the earliest form of collagen to be produced in tendon healing process is type III collagen, which has a maximum of production during the proliferative phase, and which is then gradually replaced by type I collagen in the terminal phase of the process, during the remodelling 8. However, type III collagen is weaker and is one of the causes of the increased risk of new injuries following a first damage of tendon tissue 47. In human fibroblast cultures subjected to cyclic stretching, a scenario compatible with the proliferative phase was observed, with a higher mRNA expression of type III collagen, followed by type I collagen 43. On the contrary, in our experiments an increased type I collagen mRNA level compared to type III (Fig. 4) was observed, a result allowing to speculate that our experimental model might mimic more closely the last phase of the healing process, resulting in formation of an extracellular matrix more affine to the healthy tendon tissue than to the tissue in the healing phase. This result is very promising as going in the direction of engineered tissues possessing functional biomechanical features, similar to native tissues.

The data obtained from the constructs mechanical tests go in the same direction, confirming that the construct treated with TGF‐β/AA and subjected to cyclic mechanical stretching (3DST) possesses the highest elastic modulus value, which almost doubled that of the internal control (3DSC) (Table. 1). This result confirms that the synergistic application of biochemical and mechanical stimuli is crucial for obtaining a better engineered tissue with a higher degree of matrix organization, conferring a higher elastic modulus and enhanced endurance. In fact, the mechanical stimulation itself is able to promote alignment of collagen fibres and proper organization of the extracellular matrix (Fig. 3I and J), while treatment with TGF‐β/AA promotes a higher expression of type I collagen and a lower expression of type III, resulting in the production of an abundant extracellular matrix typical of healthy tendon tissue.

## Conclusions

In this work, we present an approach for producing artificial tissue which is potentially relevant to tendon tissue engineering. Indeed, the combined provision of biochemical and mechanical stimulation promoted the expression and the production of an extracellular matrix with a collagen balance very close to that of the native tendon tissue. Furthermore, this extracellular matrix is also properly organized, with compact type I collagen fibres arranged parallel to the stretching direction, a feature that considerably increases the elastic modulus and endurance of the matrix itself. This system is therefore a good starting point for the fabrication of an engineered tendon tissue that could be further improved screening and selecting the optimal source of tenogenic stem cells and increased conditioning times.

## Conflict of interest

The authors state no conflict of interest regarding the present paper publication.

## Supporting information


**Figure S1.** Viability assay performed on 3D constructs.

**Figure S2.** Nuclear staining of 3D constructs.

**Figure S3.** Tensile testing for pristine PF (NC) and freshly seeded constructs (T0).

**Video S1.** Imposed mechanical stretching using a custom‐developed bioreactor.

## Note

1 Duty cycle is defined as the fraction of one period in which a system is active.
