# Supplementary figures and images for: Combination of biochemical and mechanical cues for tendon tissue engineering
Source: J Cell Mol Med. 2017 May 4;21(11):2711–9. doi: 10.1111/jcmm.13186 (PMC5661263; doi:10.1111/jcmm.13186)

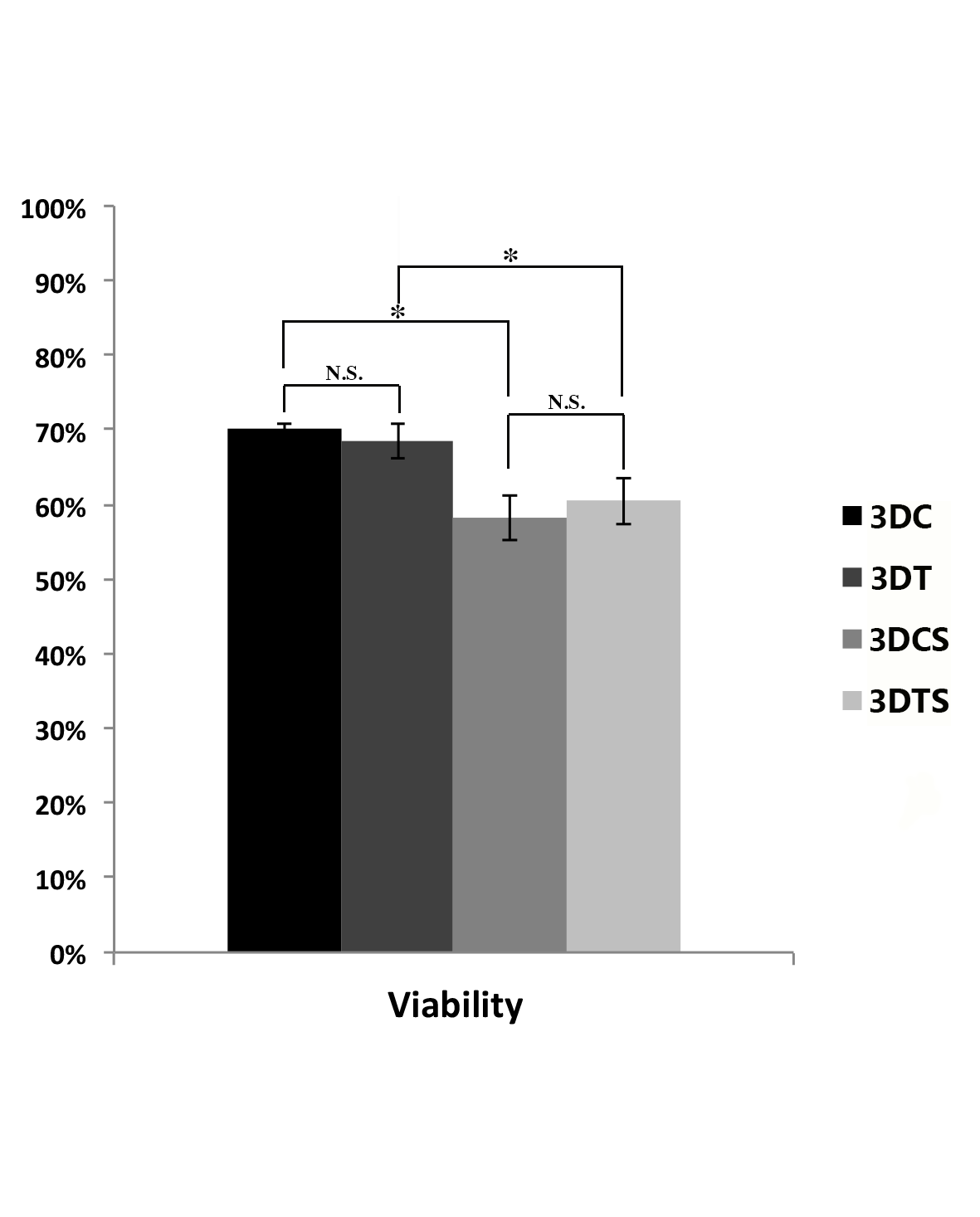

Supplement: Supplementary file 1 — Figure S1. Viability assay performed on 3D constructs. [file JCMM-21-2711-s001.tiff]

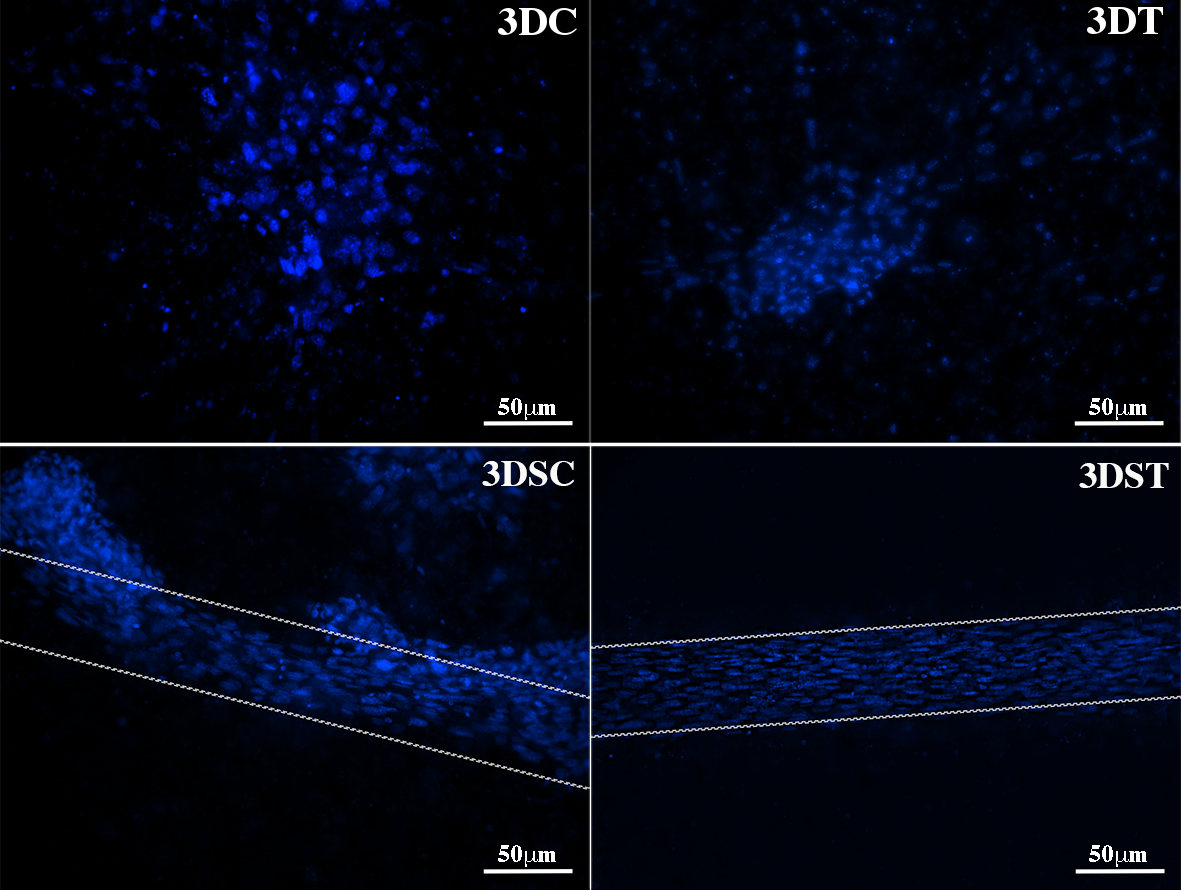

Supplement: Supplementary file 2 — Figure S2. Nuclear staining of 3D constructs. [file JCMM-21-2711-s002.tiff]

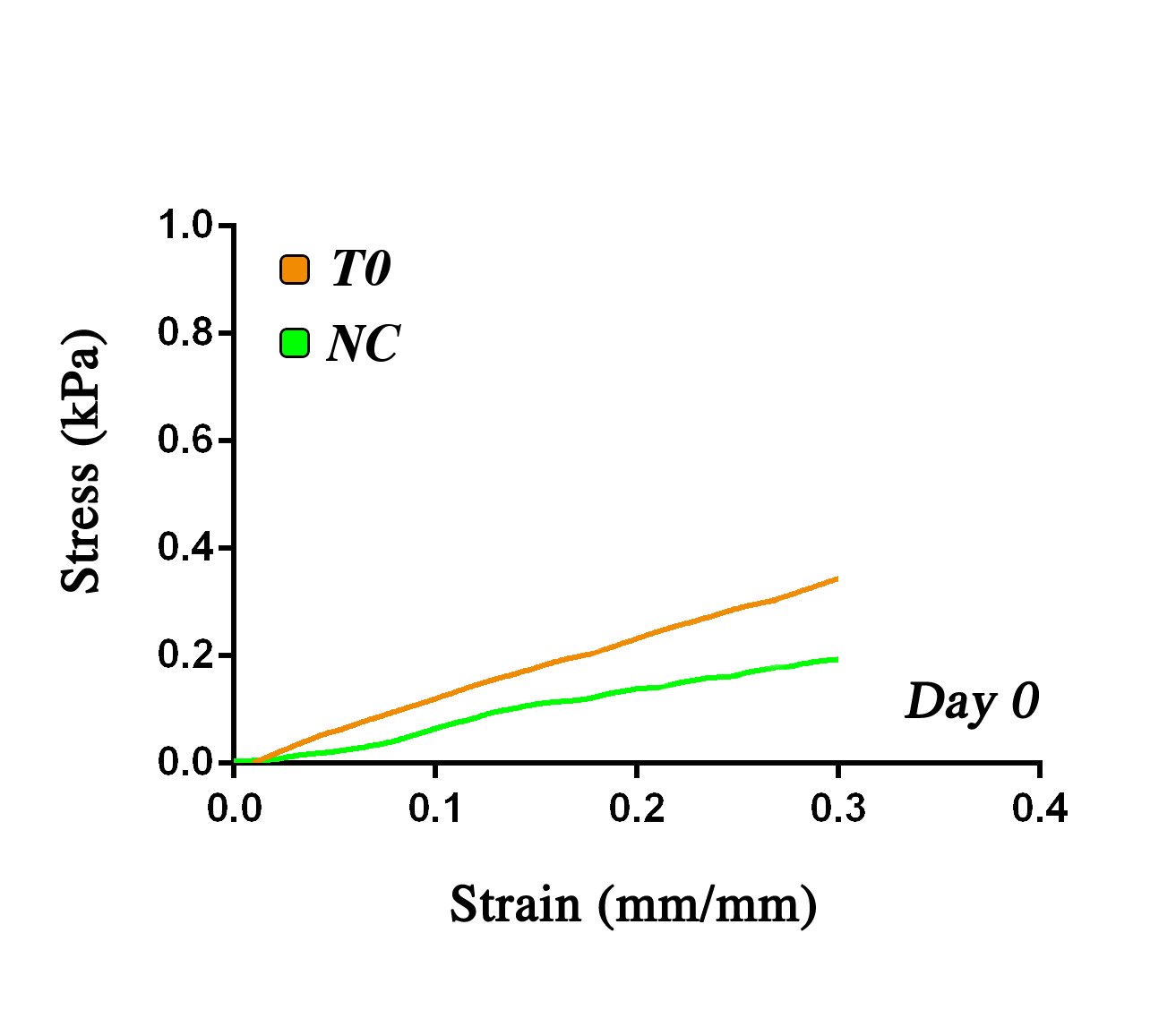

Supplement: Supplementary file 3 — Figure S3. Tensile testing for pristine PF (NC) and freshly seeded constructs (T0). [file JCMM-21-2711-s003.tiff]
